# Educational Interventions on Chronic Kidney Disease for Care Home Staff: An Empty Scoping Review

**DOI:** 10.3390/nursrep16040135

**Published:** 2026-04-13

**Authors:** Grace Crolly-Burton, Gary Mitchell, Clare McKeaveney, Stephanie Craig

**Affiliations:** School of Nursing and Midwifery, Queen’s University Belfast, Belfast BT9 7BL, UK; gcrollyburton01@qub.ac.uk (G.C.-B.); gary.mitchell@qub.ac.uk (G.M.); c.mckeaveney@qub.ac.uk (C.M.)

**Keywords:** care homes, chronic kidney disease, scoping review, education, educational interventions, aged care facilities, nursing care

## Abstract

**Background:** Chronic kidney disease (CKD) is highly prevalent among older adults, particularly those living in care homes, where early identification and effective management are essential to improving outcomes. **Aim:** This scoping review aimed to explore and map educational interventions designed to support care home staff in the prevention, assessment, and management of CKD. **Methods:** A scoping review (ScR) was conducted and guided by the Preferred Reporting Items for Systematic Reviews and Meta-analysis extension for ScR (PRISMA-ScR) checklist. A systematic search of six major databases was conducted following the Joanna Briggs Institute methodology. **Results:** A total of 6599 records were identified and 5573 titles and abstracts were screened; 10 full texts were assessed, but no studies met the inclusion criteria. **Conclusions:** This empty review highlights a significant gap in the literature and reinforces the need for targeted research to develop and evaluate training interventions for care home staff managing residents with CKD.

## 1. Introduction

Chronic Kidney Disease (CKD) is a prevalent and progressive condition with substantial global health implications [1]. It is estimated to affect approximately 843.6 million individuals worldwide, making it one of the most pressing non-communicable diseases of the 21st century [1]. CKD not only contributes significantly to morbidity and mortality but also imposes a considerable burden on healthcare systems due to its complex and resource-intensive management [2]. When CKD is not recognised and managed in a timely manner, it may progress to more advanced disease and contribute to complications including cardiovascular events, electrolyte disturbance, medication-related harm, avoidable hospitalization, and progression to kidney failure [2]. These consequences are particularly important in older adults, for whom CKD frequently coexists with frailty, multimorbidity, and polypharmacy. Earlier identification and treatment may help reduce preventable deterioration, support safer prescribing and monitoring, and potentially lessen avoidable healthcare utilization and associated costs [3]. In the United Kingdom (UK), over 1.9 million individuals have been diagnosed with CKD; however, the actual prevalence is likely higher due to the asymptomatic nature of the disease in its early stages, resulting in a significant number of undiagnosed cases [3].

CKD is defined as persistent abnormalities in kidney structure or function lasting for more than three months, with implications for an individual’s health (National Institute for Health and Care Excellence (NICE) [4]. The condition is classified into five stages based on the estimated glomerular filtration rate (eGFR) and markers of kidney damage. Early stages (CKD 1–3a) are often asymptomatic and may go unnoticed without routine screening, while more advanced stages (CKD 3b–5) are associated with a higher risk of complications such as cardiovascular disease, electrolyte imbalances, and progression to end-stage renal disease [4]. The presence of comorbidities such as hypertension, type 2 diabetes mellitus, and cardiovascular disease significantly increases the risk of CKD onset and progression [5]. These risk factors are particularly relevant in older populations, where both age-related renal decline and the accumulation of comorbid conditions contribute to increased vulnerability [6].

Care home residents are particularly at risk due to the intersection of advanced age, multimorbidity, and reduced functional capacity [7]. In the UK, the proportion of individuals requiring residential care, especially in settings offering nursing services, has steadily increased. Data from the Office for National Statistics [8] indicate that more than one-third of care home residents in England and Scotland report being in ‘bad or very bad health’. This population is therefore likely to include a significant number of individuals either diagnosed with or at risk of developing CKD. Research by Carter et al. [9] identified a high prevalence of CKD within residential care environments, with 83% of residents having CKD stage ≥ 3 when estimated using the Modification of Diet in Renal Disease (MDRD) equation and 97% when using the Cockcroft–Gault equation. This highlights the need for tailored, patient-specific management strategies that consider the unique clinical and functional profiles of older adults. The term care home refers to facilities that offer 24 h care and supervision, primarily for older adults with complex or high-level care needs [10]. This includes both nursing homes where care is delivered or overseen by registered nurses and residential care homes, which provide personal and social support without on-site nursing staff [11]. Terminology differs across countries: in Australia, such settings are commonly known as Residential Aged Care Facilities (RACFs) [12], while in the United States, the closest equivalents are nursing homes or long-term care facilities [13]. These care homes are distinct from assisted living facilities, home care services, and short-term rehabilitative skilled nursing facilities, which typically offer lower-intensity or temporary support. For the purposes of this review, care home refers to any setting that provides full-time, ongoing care whether nursing-led or residential for individuals who can no longer be safely supported in their own homes.

Effective CKD management requires early identification, regular monitoring, and timely therapeutic interventions aimed at slowing disease progression and preventing complications [14]. In care homes, much of this responsibility falls on nursing and support staff, who must possess adequate knowledge and skills in managing CKD. Their roles often include overseeing dietary modifications, monitoring fluid balance, managing polypharmacy, and recognizing signs of disease progression or complications [15]. Where training is limited, there is a risk that subtle indicators of deterioration may be overlooked, opportunities for early escalation may be missed, and routine care processes such as hydration monitoring, medication review, and referral may be suboptimal. In turn, this may contribute to avoidable complications and inefficient use of health services. Given the multifaceted nature of CKD and its frequent coexistence with other chronic conditions, multidisciplinary input is essential, involving nephrologists, general practitioners, dietitians, pharmacists, and social care professionals [16]. However, the extent to which care home staff are equipped to collaborate within such frameworks is highly dependent on the quality and availability of training they receive.

Despite the recognised importance of staff competency in CKD management, there remains a notable deficiency in targeted educational initiatives within care home settings. Research suggests that gaps in knowledge, insufficient training, limited resources, and lack of access to evidence-based guidelines act as major barriers to optimal care delivery [6,17]. In this review, structured educational intervention refers to any organised educational activity developed to improve care home staff knowledge, skills, confidence, or practice in relation to CKD, including, for example, in-service teaching, workshops, online learning, blended learning, or protocol-embedded training. Structured training interventions have demonstrated potential to enhance clinical knowledge, improve performance, and positively impact patient outcomes in other chronic disease contexts [18,19,20]. Yet, within the context of CKD in care homes, the literature remains sparse and fragmented, with limited synthesis of existing educational strategies and their effectiveness.

A scoping review was therefore considered appropriate because the field is emerging, the extent and characteristics of the literature were unclear, and the purpose of the review was to map existing evidence, identify how interventions have been designed and evaluated, and clarify gaps in knowledge. A preliminary search was undertaken to refine key terms and confirm the need for a focused synthesis on CKD educational interventions for care home staff.

This scoping review (ScR) aimed to systematically map the available evidence on educational interventions related to CKD for staff in care home settings. Specifically, the review sought to identify how CKD training for care home staff has been designed, delivered, and evaluated, and to determine what staff- or resident-level outcomes, if any, have been reported. A further aim was to identify key gaps in the literature and clarify the extent and nature of the evidence base in this underexplored area. The specific objectives of this review are:

1. Map and summarize existing educational and training interventions available to staff in care homes regarding CKD awareness, prevention, detection, and management.
2. Explore the reported outcomes of educational interventions for care home staff, including changes in knowledge, skills, confidence, and where available, impacts on resident care.
3. Identify gaps and limitations in current educational interventions for CKD care in care homes to inform future research and practice.

## 2. Materials and Methods

### 2.1. Study Design

This scoping review was conducted using the methodological framework proposed by Arksey and O’Malley [21] informed by Joanna Briggs Institute (JBI) guidance [22] for scoping reviews and reported in line with the Preferred Reporting Items for Systematic Reviews and Meta-Analyses extension for Scoping Reviews (PRISMA-ScR) checklist [23]. The review process included formulating a research question, identifying relevant literature through systematic searches, developing and applying eligibility criteria, and selecting and screening relevant studies. A scoping review protocol was registered with the Open Science Framework (OSF) (OSF DOI Registration No: 10.17605/OSF.IO/BMJFK; available at https://osf.io/bmjfk) accessed on 16 April 2025. Please see Supplementary Materials for a copy of the PRISMA-ScR checklist.

### 2.2. Search Strategy

The search strategy was developed collaboratively by the review team, with input from a subject librarian to ensure comprehensiveness and relevance. A preliminary search was also conducted using Google Scholar to help refine key terms and identify initial sources. A Population, Exposure, Outcome (PEO) framework was used to structure the search strategy and key search concepts for this review [24].

- Population (P): individuals living with chronic kidney disease (CKD), identified through CKD-specific terminology (e.g., “chronic kidney disease,” “renal insufficiency,” “kidney failure”).
- Exposure (E): settings providing residential support, including nursing homes, care homes, and long-term care facilities.
- Outcome (O): educational or training interventions designed to improve CKD knowledge, awareness, or care practices among staff working in these settings.

Search terms were deliberately limited to CKD-specific language (e.g., “chronic kidney disease,” “renal insufficiency,” “kidney failure”) rather than broader comorbidities such as hypertension or diabetes. This ensured the inclusion of studies explicitly designed as CKD management education rather than generic long-term condition training. The search dates were from 3 September 2025 to 31 December 2025 for MEDLINE (via PubMed), Scopus, CINAHL, EMBASE, Web of Science, and PsycINFO. The search included materials published from database inception until 31 December 2025, with no date restrictions applied. Searches included both peer-reviewed and grey literature sources. Grey literature was considered important in this applied field because relevant evidence may be reported in theses, dissertations, organisational reports, service evaluations, or other non-indexed documents. In addition to database searching, supplementary searching included screening the reference lists of relevant papers and the use of Google Scholar during the preliminary search stage to refine key terms and identify potentially relevant material. However, no additional eligible records were identified through citation searching or other non-database sources. A full and reproducible example of the MEDLINE search strategy, including Boolean operators and subject headings (e.g., MeSH, EMTREE), is presented in Table 1. This strategy was tailored to each database by adapting subject headings, field labels, and search syntax to the requirements of the individual platform, while retaining the same core concepts and logic across all databases.

Title and abstract screening were independently conducted by two reviewers (G.C.B and S.C.), with disagreements resolved by a third reviewer (G.M.).

### 2.3. Eligibility Criteria

To address the review question, the search considered empirical research on CKD educational interventions in care home settings, as well as relevant grey literature, including theses, dissertations, and other non-peer-reviewed outputs. Eligible settings included care homes, nursing homes and residential facilities. Educational interventions of any type were eligible, including in-person, online, and self-directed approaches. Studies were excluded if they did not investigate an educational intervention, were conducted in settings other than care homes (e.g., hospitals, community clinics, primary care settings, or skilled nursing facilities), or did not explicitly address CKD management. Articles written in any language were considered, and no time restrictions were applied. Table 2 presents the full inclusion and exclusion criteria used in this ScR.

### 2.4. Study Selection

Covidence software (https://www.covidence.org/, accessed on 3 September 2025) was used to facilitate duplicate removal, literature screening, and data extraction. Title and abstract screening were conducted independently by G.C.B and S.C, with both reviewers screening 100% of the records. Conflicts at this stage were resolved through discussion with a third reviewer G.M. No formal inter-rater agreement statistic was calculated.

### 2.5. Data Extraction and Analysis

As no studies met the inclusion criteria following full-text screening, no data extraction was conducted.

## 3. Results

**Figure 1:**
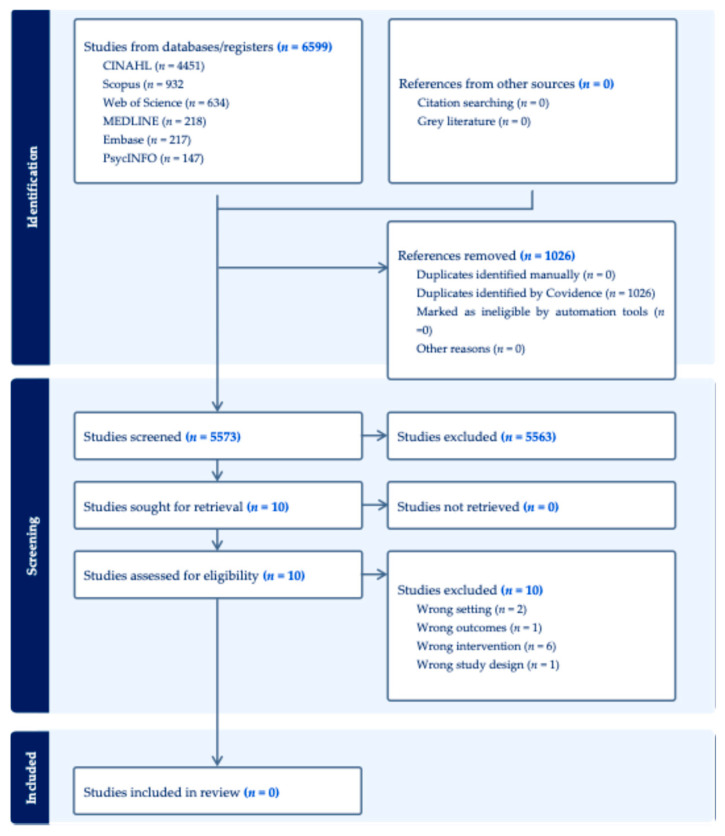
PRISMA-ScR flowchart. PRISMA: Preferred Reporting Items for Scoping Reviews and Meta-Analyses.

A total of 6599 papers were identified through the database searches. After removing 1026 duplicates, 5573 titles and abstracts were screened. Ten records were retrieved for full-text assessment; however, none met the inclusion criteria. Common reasons for exclusion at full-text review were wrong intervention (*n* = 6), wrong setting (*n* = 2), wrong outcomes (*n* = 1), and wrong study design (*n* = 1), as illustrated in the PRISMA-ScR flowchart in Figure 1.

As a result, no studies were eligible for data extraction. Despite the initial volume of literature retrieved, this review identified a clear gap in the published evidence regarding educational interventions specifically designed to support care home staff in the prevention, assessment, and management of CKD.

## 4. Discussion

This scoping review aimed to map and synthesise existing educational interventions aimed at supporting care home staff in the assessment, prevention, and management of CKD. Despite an extensive search, no studies met the inclusion criteria, resulting in an empty review. This lack of evidence highlights a critical gap, and, considering the growing prevalence of CKD within the care home population this gap is concerning. Empty reviews are not uncommon [25,26]. For example, the Cochrane Database of Systematic Reviews included 4320 systematic reviews, of which 376 (8.7%) were classified as empty, having found no studies that met the inclusion criteria [27].

CKD is highly prevalent among residents of nursing homes, estimates vary depending on study context and diagnostic thresholds, for example, in a study by McClellan et al. [28] it was found that approximately half of the nursing home population included had CKD, with the majority of those presenting with moderate disease (stage 3a and stage 3b). To further support this claim, a study conducted by Eren et al. [29] reported prevalence of around 40% among long term care residents, with a substantial proportion being stage 3 or above. CKD is undoubtably a significant burden to the vulnerable care home resident population, these figures underscore the need for early detection and management strategies in care home settings.

While no studies were identified that focused specifically on care home settings, literature from broader healthcare contexts suggests that CKD-related educational interventions may improve healthcare providers’ knowledge, clinical performance, communication, and patient outcomes [6,30]. However, these findings should be interpreted cautiously in relation to care homes. Care home settings differ from acute, primary care, and specialist renal services in important ways, including staffing mix, access to on-site clinical expertise, competing workload demands, turnover, and opportunities for continuing professional development. As such, evidence from other settings is useful for contextual reflection but cannot be assumed to translate directly to the care home environment.

The absence of eligible studies in this review may reflect several underlying factors. Structural and organisational barriers including complex ethical approvals, limited research capacity within care homes, heavy staff workloads, and gatekeeping are well recognised as constraints on conducting studies in these settings [31,32]. In addition to these research barriers, the everyday realities of care home functioning can act as obstacles to implementing and sustaining educational interventions. Staff shortages and high workload demand limit opportunities for dedicated training time, while high turnover undermines continuity and long-term impact. Furthermore, competing clinical priorities, limited resources, and restricted access to continuing professional development are recurring challenges in nursing home contexts [33,34]. These contextual barriers are relevant considerations when interpreting the evidence gap identified in this review. However, the present review cannot determine whether they directly caused the absence of CKD-specific educational interventions in care homes. Rather, they offer a plausible contextual explanation for why intervention development, evaluation, and publication in this setting may be limited, and they highlight the need for any future programmes to be designed with feasibility and sustainability in mind [35]. Taken together, these findings suggest that the absence of studies should not necessarily be interpreted as an absence of educational need, but rather as an indication that CKD-specific education for care home staff remains poorly developed, under-evaluated, or insufficiently visible within the published literature.

It is also possible that CKD-specific training has been subsumed under broader educational programes targeting multimorbidity, diabetes, or cardiovascular disease, and therefore not captured within our eligibility criteria. A critical reflection on our own methodological choices is therefore warranted. Although a preliminary search was undertaken to refine the review question and search terms, the absence of eligible studies suggests that the field is either extremely underdeveloped or that relevant CKD content is embedded within broader educational interventions not indexed or described in CKD-specific terms. Our decision to limit inclusion to studies explicitly addressing CKD-related education in care homes was intended to maximize practical applicability to UK practice, but it may have excluded studies in which CKD was addressed as part of broader chronic disease education. While we believe this narrow focus is justified, it is important to acknowledge that it may have contributed to the absence of eligible studies. Taken together, the evidence gap identified in this review should not be interpreted as an absence of educational need. Rather, it suggests that CKD-specific education for care home staff is either underdeveloped, under-evaluated, or insufficiently visible in the published literature. This has important implications, as it indicates a disconnect between the likely clinical importance of CKD in care home populations and the current state of staff-focused educational research. The gap therefore points not only to a missing body of evidence, but also to a missed opportunity to equip care home staff with condition-specific knowledge in a setting where early recognition, monitoring, and escalation may be particularly important. Future reviews may wish to adopt a broader scope to determine whether transferable lessons can be drawn from more general educational interventions.

### 4.1. Limitations

This review is not without limitations. First, no studies met the inclusion criteria, which prevented the synthesis of evidence regarding effective educational interventions to support the management of CKD in care homes. Second, our search strategy was limited to CKD-specific terminology; as such, interventions focused more broadly on chronic disease risk factors (e.g., hypertension, diabetes) may have been overlooked, despite their potential conceptual relevance. Third, the review retained a narrow focus on care home settings and excluded studies conducted in skilled nursing facilities in order to align more closely with UK care models; this may have reduced opportunities to capture potentially transferable evidence from other international long-term care contexts. Finally, although grey literature sources such as theses and dissertations were eligible, the review did not include all possible forms of non-indexed or practice-based material, such as local service reports or organisational training documents. Relevant educational activity may therefore remain underrepresented in the published and retrievable evidence base. Finally, although grey literature sources such as theses and dissertations were considered potentially relevant, the review did not identify any retrievable grey literature and did not include all possible forms of non-indexed or practice-based material, such as local service reports or organisational training documents. It is therefore possible that relevant educational initiatives relating to CKD in care home settings are being delivered in practice but have not been formally evaluated, published, or made retrievable through academic or grey literature sources.

### 4.2. Future Directions

Although evidence is lacking, several priorities emerge for future research. First, intervention development should focus on CKD content most relevant to routine care home practice, including recognition of declining renal function, interpretation and escalation of abnormal renal indicators, hydration and fluid balance, medication safety in the context of renal impairment, dietary considerations, and timely referral or communication with primary and specialist services. Second, future studies should examine delivery models that are realistic within care homes, such as brief in-service education, case-based teaching, digital micro-learning, or blended approaches embedded within existing staff development structures. Third, intervention studies should report implementation detail clearly, including staff group targeted, duration, frequency, content, mode of delivery, feasibility, acceptability, and barriers to uptake in contexts characterised by workload pressure, staff turnover, and limited protected training time. Finally, evaluations should move beyond immediate knowledge gain to examine outcomes with direct relevance to CKD care in care homes, such as staff confidence in recognising deterioration, quality of monitoring and escalation, medication-related safety, potentially avoidable hospital transfer, and resident-centred outcomes where feasible.

## 5. Conclusions

This review found no eligible studies examining CKD-related educational interventions for care home staff. This empty-review finding should not be interpreted as an absence of educational need; rather, it suggests that CKD-specific education in care home settings is underdeveloped, under-evaluated, or insufficiently visible in the published literature. Given the high burden of CKD among care home residents, this gap is important and points to the need for context-sensitive intervention development and evaluation. Future research should focus on designing and testing feasible CKD educational interventions for care home staff that reflect the organisational realities of long-term care and evaluate both staff- and resident-level outcomes.

## Figures and Tables

**Table 1:** Search Strategy Example, adapted for Ovid Medline.

Key Words (P)		Key Words (E)		Key Words (O)
exp Kidney Diseases, Chronic/		exp Nursing Homes/		exp Education/
OR		OR		OR
exp Renal Insufficiency, Chronic/		exp Homes for the Aged/		exp Education, Nursing/
OR		OR		OR
exp Kidney Failure		exp Intermediate Care Facilities/		exp Education, Professional/
OR		OR		OR
exp Renal Replacement Therapy/	AND	exp Long-Term Care/	AND	exp Inservice Training/
OR		OR		OR
exp Renal Dialysis/		exp Residential Facilities/		exp Health Education/
OR		OR		OR
chronic kidney disease.mp.		care home*.mp.		exp Program Evaluation/
OR		OR		OR
CKD.mp.		nursing home*.mp.		educational intervention*.mp.
OR		OR		OR
renal insufficiency.mp.		residential home*.mp.		training.mp.
				OR
				Professional development.mp
				OR
				CPD.mp.
				OR
				staff development.mp.
				OR
				in service training.mp.
				OR
				program*.mp.
				OR
				e-learning.mp.
				OR
				self-directed learning.mp.

**Table 2:** Inclusion and Exclusion Criteria.

Inclusion	Exclusion
Any output reporting any studies design including but not limited to trials, observational research, qualitative methodologies	Hospital, acute, community or any settings other than care homes
Literature reviews, theses, dissertations	Commentary papers
Studies that include an educational intervention or training	Non care home staff
Articles written in any language	Studies that do not include an educational intervention
No time restrictions	

## Data Availability

No new data were created or analyzed in this study.

## Supplementary Materials

The following supporting information can be downloaded at https://www.mdpi.com/article/10.3390/nursrep16040135/s1: Supplementary File S1: Preferred Reporting Items for Systematic reviews and Meta-Analyses extension for Scoping Reviews (PRISMA-ScR) Checklist.

## Public Involvement Statement

No public involvement in any aspect of this research.

## Guidelines and Standards Statement

This manuscript was drafted against the PRISMA extension for Scoping Reviews (https://www.equator-network.org/reporting-guidelines/prisma-scr/ accessed on 10 May 2025).

## Abbreviations

The following abbreviations are used in this manuscript:

CKD	Chronic Kidney Disease
NICE	National Institute for Health and Care Excellence
UK	United Kingdom
eGFR	Estimated Glomerular Filtration rate
